# Prevalence of depression among adolescents in rural communities of Mexico

**DOI:** 10.7189/jogh.15.04238

**Published:** 2025-10-17

**Authors:** Lina Díaz-Castro, Gerardo Andrés Vega-Rosas, Gerardo Bernabe Ramírez-Rodríguez, Hector Cabello-Rangel, Kurt L Hoffman

**Affiliations:** 1 National Institute of Psychiatry Ramón de la Fuente Muñiz, Department of Epidemiological and Psychosocial Research, Mexico City, Mexico; 2 National Institute of Psychiatry Ramón de la Fuente Muñiz, Sub-directorate of Clinical Research, Mexico City, Mexico; 3 National Institute of Psychiatry Ramón de la Fuente Muñiz, Sub-directorate of Clinical Research, Mexico City, Mexico; 4 Psychiatric Hospital Fray Bernardino Álvarez, Department of Research, Mexico City, Mexico; 5 Autonomous University of Tlaxcala – Cinvestav, Animal Reproduction Research Center, Tlaxcala, Mexico

## Abstract

**Background:**

Rural communities face challenges to mental health care, including cultural barriers and lack of accessibility. There is a general scarcity of data on the mental health of adolescents in rural regions of Mexico. The aim of the present study was to determine the prevalence of depression in adolescents and young adults in a rural Mexican community.

**Methods:**

A cross-sectional observational study was conducted from April to September 2023, of individuals aged 15–25 of rural communities of San Luis Potosí, Mexico. Stratified sampling (n = 1057) ensured gender/age representation. Participants received two psychoeducation sessions, followed by a Spanish-language the 9-item Patient Health Questionnaire (PHQ-9) scale to quantify depressive symptoms. Mann-Whitney U and Fisher Exact tests were used to compare continuous and categorical variables, respectively. Principal component analysis was used to identify the most important items associated with probable clinical depression.

**Results:**

Sixty-one percent of the participants exhibited depressive symptoms, 23% met criteria indicating a probable diagnosis of major depressive disorder, and 29% reported suicidal ideation. A probable diagnosis of major depressive disorder was significantly more frequent in female subjects, those with a personal history or familial antecedents of depression, and those that experienced suicidal ideation. Most endorsed PHQ-9 items were sleep disturbances and feeling tired or low on energy, and females had significantly higher PHQ-9 scores than males. Principal Component Analysis indicated a single factor that explained 55% of the variance and encompassed all nine items, while suicidal ideation was additionally associated with a second factor that accounted for an additional 10% of variance.

**Conclusions:**

There is a significant depression burden among rural Mexican youth. Psychoeducation may help adolescents identify past and present episodes of depression in themselves as well as in their family and peers. Targeted mental health services in rural communities are necessary to confront disparities in mental health care.

Depressive disorders (DD), as defined by the American Psychiatric Association [1], are among the most prevalent chronic mental disorders globally [2,3]. These conditions affect approximately 322 million people, representing 4.4% of the world's population, with a significant proportion residing in the Americas, including Mexico [4]. The prevalence of DD among adolescents and young adults is particularly concerning, having increased by more than 50% in recent decades [5]. In Mexico, this age group alone accounts for 426 Disability-Adjusted Life Years (DALYs) per 100 000 people [5]. A study of Mexican adults reported an overall prevalence of DD of 4.5%, with a higher prevalence in women (5.8%) compared to men (2.5%); these statistics are similar to those reported globally [6,7]. Depression is also associated with elevated risks of chronic physical conditions such as arthritis, chronic pain, heart disease, stroke, hypertension, diabetes mellitus, asthma, chronic lung disease, peptic ulcer and cancer, early mortality, and an increased suicide risk of approximately 15% [8,9].

Adolescence represents a critical period for the onset of depressive symptoms, which constitute the leading cause of morbidity and disability in this age group [10–12]. Globally, approximately 34% of this population presents elevated symptoms of depression, and there has been a notable rise in the prevalence of depressive disorders in low- and middle-income countries over the past three decades [13].

Studies of Mexican adolescents carried out prior to the year 2015 indicated that the prevalence of clinical depression in adolescents was in the range of 8–17% [14]. Adolescents that suffer from clinical depression have five times greater odds of attempting suicide [15], which is the second leading cause of death in this demographic (six deaths per 100 000; violent deaths by firearms rank first, with a rate of 15 per 100 000 deaths [5]). The lifetime prevalence of suicide attempts in Mexican adolescents has risen dramatically in recent years, from 1.1% in 2012 to 3.9% in 2018 [15]. Recent surveys reported that the prevalence of suicidal ideation in a sample of Mexican adolescents was around 15% [16], and around 19% of adolescents were at high risk of suicide [17,18]. Suicide attempts and ideation are markedly more prevalent in adolescent Mexican girls compared to boys [15–17].

In Mexico, mental health services are predominantly concentrated in urban centres [19]. Studies of rural Mexican adult populations show that low socioeconomic status and familial stress, limited educational opportunities and access to mental health care, language barriers, absence of a spouse due to emigration, and even agricultural pesticide exposure can impact significantly on the prevalence of depression in rural communities [20–22]. Unfortunately, studies of depression in rural adolescent populations of Mexico are scarce: we were able to find only one such study, carried out during the year 2019 in four rural indigenous communities in the state of Chiapas [23]. In that study, the prevalence of mild to severe depression in adolescents of ages 14–20 was reported to be 20.6%; severe depression was reported to be 3%. In recent studies of adolescents and young adults carried out in more urban areas, the prevalence of depressive symptoms (moderate to extremely severe) was reported to be 34%, while the prevalence of extremely severe depression was approximately 12% [16,24].

Rural and indigenous communities face unique challenges for the provision of mental health care. Often there are significant barriers to accessing care in these communities, and this problem is compounded by social, economic, and cultural barriers [25]. These and other challenges can exacerbate mental health problems in these areas. For example, in Canada, indigenous people showed higher psychological distress scores, and higher prevalence of suicidal ideation (16.8 *vs*. 9.2%) compared to non-indigenous groups; this difference was largely accounted for by socioeconomic factors [26]. Likewise, a study of South American indigenous populations revealed an alarmingly increased suicide rate compared to the general population [27].

In Mexico, information on the prevalence of depression in adolescents and young adults of rural and indigenous communities is sparse. In order to formulate policy for effectively addressing the inequities in mental health care between urban and rural/indigenous populations, it is necessary to obtain data on the prevalence and severity of psychiatric symptoms in these populations. Therefore, the aim of the present study was to investigate the prevalence and specific characteristics of depressive symptoms among adolescents and young adults living in a rural Mexican community that has a large indigenous population. Considering that previous research has reported gender differences in the prevalence of depression, we predicted that the expression and severity of depression symptoms would differ according to gender in this population.

## METHODS

This cross-sectional study was conducted between April–September 2023 in the communities of the Municipality of Ciudad Fernández, in the State of San Luis Potosí, Mexico. Data were collected by means of questionnaires applied as part of an ongoing project (Project number S.736, funding agency: Gonzalo Río Arronte Foundation I.A.P.) to assess the prevalence of depressive symptoms in adolescents and to implement a telepsychiatry service in rural regions of Mexico.

### Setting

San Luis Potosí is one of the Mexican states with the highest number of indigenous language speakers. With a population of 2 822 255 people, more than 17% (approximately 480 000 individuals) fall within the 15–24 age group. Additionally, over 23% of the state's population, an estimated 630 604 individuals, is classified as indigenous [28].

Of the 58 municipalities in San Luis Potosí, the Huasteca Region contains the largest indigenous population. Although the initial plan was to conduct this study in this region, logistical constraints related to infrastructure and security necessitated selecting a municipality in the Middle Zone of the state. This region was chosen due to its relatively high number of indigenous and young residents. Consequently, the communities within the municipality of Ciudad Fernández were identified as the study participants. Ciudad Fernández consists of the municipal seat, 13 ‘ejidos’ (large, communally farmed tracts of land), and 19 ranches. According to the 2020 Mexican census by the National Institute of Statistics and Geography [28], the municipality had a population density of 92.8 inhabitants per square kilometre, distributed across 97 localities. The municipal seat, Ciudad Fernández, is the most populous locality, with 36 275 inhabitants, representing 17% of the state's total population. Of these, 48.7% are male and 51.3% are female, with an average age of 28 years.

### Participants and sample size

The study participants consisted of 1057 young individuals aged 15–25 years, residing in various communities within the municipality of Ciudad Fernández, located in the state of San Luis Potosí, Mexico. The sample size was determined a priori using G*Power 3.1 to detect a small to medium effect size (Cohen’s d = 0.30) with 80% power at α = 0.05. An estimated 20% non-response rate was accounted for, resulting in a target recruitment of 800 participants [29].

A stratified sampling strategy was applied to guarantee the inclusion of diverse sociocultural contexts within the rural and indigenous communities. The stratification was based on two key variables:

1) type of community (rural mestiza *vs*. rural indigenous)

2) the ethnocultural composition, using official census data and community indicators of the local health districts.

The communities were selected intentionally in collaboration with the regional health authorities and local leaders, prioritising zones with high social vulnerability levels, limited access to mental health services and different population densities of indigenous people. Within each selected community, a convenience sample was used, recruiting participants between the ages of 15–25 within schools, health centres, and local youth programmes. Local community health personnel and school authorities helped identify the eligible participants and facilitated the procedures of informed consent.

### Procedure

Data were collected by persons trained as ‘Mental Health Promoters.’ These professionals conducted two outreach and psychoeducation sessions that each had a duration of approximately 45-minute. These sessions were held in various community spaces that were frequently visited by young people, mainly schools, sports facilities, gyms, entertainment centres, and public squares. During the first session, a brief explanation of the purpose of the study was presented to potential participants, and then they were invited voluntarily and anonymously to participate in the study. Young individuals (15–25 years of age) who agreed to participate provided written informed consent or assent, as appropriate. Basic sociodemographic data were collected from each participant (identified only by their initials), in addition to a survey designed to assess the participants’ general knowledge and perceptions of the symptoms of depression (these data will be published in a separate study). During the second session, Mental Health Promotors provided information to the participants on the characteristics of symptoms of depression, and how to identify the presence of such symptoms. Following this session dedicated to psychoeducation, the survey to assess general knowledge and perceptions of depression was again administered. Finally, the 9-item Patient Health Questionnaire (PHQ-9) was applied to assess the prevalence and severity of depressive symptoms in this sample population. Three ad hoc questions were added to the end of the PHQ-9, having to do with:

1) whether the participant considered that at some time in the past he/she had suffered from depression

2) if a family member suffered from depression

3) what was the familial relationship of the depressed family member (mother, father, etc.).

All collected data were entered into an Excel database, ensuring proper identification for each questionnaire and guaranteeing that the identity of each participant was protected and only the principal investigator had that information under her protection. Subsequently, the data were analysed using GraphPad Prism 10.0.2 software, (San Diego, California, USA) for macOS statistical software to identify patterns and trends.

### Instruments and measurements

#### Sociodemographic questionnaire

The questionnaire collected the following variables: age, gender, place of residence, educational attainment, academic year, marital status, and occupational activity. Participants were also asked whether they spoke an indigenous language. Additionally, the same sociodemographic information was gathered for the primary economic provider in the participant's family, along with details regarding the total family income.

#### PHQ-9: patient health questionnaire

The PHQ-9 is a validated instrument for the Mexican population, designed to detect depressive disorders based on the Diagnostic and Statistical Manual of Mental Disorders, version IV (DSM-IV)criteria. It evaluates the severity of depression and monitors symptom changes over time. The questionnaire includes nine Likert-type items (scored 0–3) that reflect experiences from the past two weeks [30]: a score of 0 corresponds to ‘on none of the days’; 1, ‘some days’; 2, ‘more than half the days’; and 3, ‘almost all days.’ The Spanish version of the PHQ-9 demonstrated comparable sensitivity (87%) and specificity (88%) to the original version [31]. The PHQ-9 total score can range from 0–27, and the following cutoff-based severity levels have been applied for this instrument:

1–4: minimal depression

5–9: mild depression

10–14: moderate depression

15–19: moderately severe depression

20–27: severe depression.

Scores on the PHQ-9 have also been used to screen for individuals with a probable diagnosis of Major Depressive Disorder (MDD), according to the following criteria: at least five items of the PHQ-9 rated as ‘more than half the days’ (score of 2) or ‘nearly every day’ (score of 3) with at least one of these items being item 1 (depressed mood) or item 2 (loss of interest/pleasure).

### Statistical analysis

Descriptive analyses were conducted to summarise the sociodemographic characteristics of the sample and the distribution of PHQ-9 scores. Normality tests failed to support a normal distribution of the PHQ-9 scores; therefore, Mann-Whitney U test was applied to compare PHQ-9 item and total scores between female and male participants, and Fisher Exact test was applied to compare proportions of participants that endorsed the most severe level (score of 3) of each item. The Biserial Correlation was calculated as an effect size corresponding to Mann-Whitney U comparisons, and Cohen’s h was calculated for comparisons of proportions. We considered effect sizes of 0.2 as small, 0.5 as medium and 0.8 as large. Principal Components Analysis (PCA) was used to explore the underlying structure of the PHQ-9 in this population, aiming to assess dimensionality and identify latent constructs that may shed light on the bases of depressive symptom patterns. Additionally, Spearman’s correlation test was used to assess the relationships between sociodemographic factors and depressive symptoms (individual PHQ-9 item scores, and PHQ-9 total score). JASP software v.0.19.3 (freely available online from the University of Amsterdam, Netherlands) and Prism version 10.0.2 for macOS (GraphPad Software, San Diego, CA, USA) were used for data analysis and for preparing the graphs. The significance level was set at *P* = 0.05.

## RESULTS

### Sociodemographic characteristics

A total of 1057 participants were recruited, of which 932 surveys were obtained. The majority of the participants were female (60.5%), with an average age of 16.6 years (range 15–25 years, standard deviation (SD) = 1.5); male and female participants did not differ significantly with respect to age. Seven participants reported speaking an indigenous language; of these seven participants, five were male. Information on the primary caregiver was available for 806 participants: 65.5% of participants reported having a female primary caregiver, (n = 528), 32.6% of caregivers were male (n = 263). The total PHQ-9 score was significantly greater for subjects that had a female primary caregiver, although the effect size was small (Mann-Whitney U test, *P* < 0.05; Rank Biserial Correlation = 0.18). The average caregiver age was 43.2 years (15–81 years, SD = 7.9). The main occupations reported by the caregivers were: 38.6% had formal employment, 28.5% were housewives, 25.9% were self-employed, 5.3% were students, 0.7% were employed for other reasons, 0.7% were retired, and 0.3% were unemployed for health reasons. Household income was reported by 300 participants, with an average income of 10 634 Mexican pesos, of which a mean of 3.8 ± 1.5 (SD = 1.5) individuals depended on this income (**Table 1**). There was no significant relationship between PHQ-9 scores and age of caregiver or household income (Spearman’s Correlation test, data not shown).

**Table 1:** Sociodemographic characteristics of the sample

Variable	n	%
Gender		
*Male*	368	39.5
*Female*	564	60.5
Indigenous language speakers	7	0.66
Caregivers		
*Male*	263	32.6
*Female*	528	65.6
*Both*	15	1.9
High school level		
*1st semester*	15	1.4
*2nd semester*	477	45.3
*4th semester*	225	21.4
*6th semester*	262	7.0
Occupational activity		
*Student*	828	86.5
*Student and employee*	50	5.2
*Student and self-employed*	54	5.64
*Student and housewife*	10	1.0

### Depression prevalence and severity

Among the 1057 participants, 768 completed the entire PHQ-9 assessment. Of these, 472 participants (61.5%) reported having some symptoms of depression (total score greater than 4); 33% (252 participants) reported moderate to severe symptoms (total score greater than 9); and 7% (54 participants) reported experiencing severe depressive symptoms (total score greater than 19). Based on their PHQ-9 scores, 23% (179 out of 768) of the participants met the criteria for a probable diagnosis of MDD. A probable diagnosis of MDD was more frequent in women (127 out of 456, 28%) compared to men (50 out of 306, 16%; Fisher exact test: *P* < 0.001; Cohen’s h = 0.28).

With regards to the three ad hoc questions that were included at the end of the PHQ-9, 52% (388 out of 742) of the participants responded in the affirmative to the question:

‘In the past, have you had several of these symptoms (such as feeling sad, loss of interest, etc.), at the same time and with an intensity and duration such that you would say that you were depressed?’

Of those subjects that reported suffering from depression in the past, 34% (134 out of 388) currently fulfilled the criteria for a probable diagnosis of MDD. By contrast, only 10% (36 out of 354) of subjects that did not have prior depressive episodes fulfilled those criteria (Fisher exact test: *P* < 0.001, Cohen’s h = 0.61). Past symptoms of depression were more frequently reported by female participants than by males (264 out of 438, 60%, compared to 122 out of 299, 41%; Fisher exact test *P* < 0.001, Cohen’s h = 0.39).

With regards to familial depression, 28% (196 out of 710) of the participants indicated that at least one family member suffered from depression, with the mother being the most frequently mentioned family member. Familial antecedents of depression were more frequent in those subjects that reported having past episodes of depression (150 out of 369; 41%) compared to those that did not (45 out of 335, 13%; Fisher exact test *P* < 0.001; Cohen’s h = 0.63), and were more frequently reported by female participants (135 out of 411, 33%) compared to male participants (59 out of 293, 20%; Fisher exact test; *P* < 0.001; Cohen’s h = 0.29). Familial antecedents of depression were also associated with elevated PHQ-9 total scores (Mann-Whitney U, *P* < 0.001; Rank Biserial Correlation = 0.54) and greater scores on each of the individual PHQ-9 items (Mann-Whitney U, *P* < 0.001; Rank Biserial Correlation ranged from 0.28–0.44). Familial antecedents of depression were also significantly associated with a current probable diagnosis of MDD: 33% (64 out of 196) of those that had family members that suffered from depression, also fulfilled criteria for a probable MDD diagnosis. By contrast, a probable MDD diagnosis was present in only 19% (96 out of 514) of those subjects that did not have family antecedents of depression (Fisher exact test, *P* < 0.001; Cohen’s h = 0.32).

When each of the items in the instrument were analysed independently, it was found that items three (sleep disturbance) and four (tiredness) were most frequently endorsed: 28% (219 out of 768) and 30% (235 out of 768) of the participants respectively reported sleep disturbances or tiredness as occurring on at least half of the days of the previous two weeks. Female participants more frequently reported experiencing items 2–9 as occurring on ‘almost all days’ compared to male subjects, with item five (appetite changes) demonstrating the largest effect size (**Table 2**). Twenty-nine percent (222 out of 768) of all respondents reported having thoughts of suicide or self-harm (item nine), and such thoughts of suicide were more frequent in those individuals with family antecedents of depression compared to those with no family antecedents (Fisher exact *P* = 0.01; Cohen’s h = 0.21), or with a current probable MDD diagnosis compared to those that did not complete criteria for a probable MDD diagnosis (120 out of 179, 67% *vs*. 102 out of 589, 17%; Fisher exact test *P* < 0.001; Cohen’s h = 1.06), or with suffering from depression in the past compared to those that did not have a past history of depression (163 out of 388, 42% *vs*. 52 out of 354, 15%; Fisher exact test *P* < 0.001, Cohen’s h = 0.62). Five percentand 4.6% of participants reported experiencing these thoughts on at least half of the days or almost every day, respectively. Very frequent thoughts of suicide (almost every day) were more frequently reported by women (6%) compared to men (2%), although the effect size of this comparison was small (**Table 2**).

**Table 2:** Gender differences in PHQ-9 individual item and total scores

PHQ-9 Item	Total*	Women*	Men*	Fisher Exact statistic*	Cohen’s h (effect size)*	Mann-Whitney U statistic†	Z score†	Biserial Rank Correlation (effect size)†
1 (lack of interest)	0.10	0.11	0.08	0.10	0.10	58 500	4.031	0.146
2 (hopelessness)	0.07	0.09	0.04	0.006	0.21	54 928	5.403	0.199
3 (sleep disturbance)	0.16	0.20	0.10	0.0002	0.28	53 882	5.617	0.203
4 (tiredness)	0.14	0.16	0.10	0.025	0.18	57 357	4.469	0.161
5 (appetite changes)	0.11	0.16	0.05	<0.00001	0.37	56 466	4.759	0.172
6 (feelings of failure)	0.11	0.13	0.08	0.012	0.16	57 798	4.261	0.154
7 (difficulty concentrating)	0.10	0.12	0.06	0.008	0.21	60 604	3.299	0.120
8 (motoric alterations)	0.05	0.07	0.02	0.006	0.25	64 098	2.139	0.078
9 (suicidal ideation)	0.04	0.06	0.02	0.01	0.21	59 964	4.138	0.150
PHQ-9 total score	-	-	-	-		52 362	5.855	0.212

PHQ-9 – 9-item Patient Health Questionnaire

*Proportions of subjects that responded ‘on almost all days’ for the corresponding items; Fisher Exact statistic and Cohen’s h effect size correspond to comparisons of these proportions between male and female subjects.

†Mann-Whitney U statistics comparing female and male PHQ-9 item scores and total scores. For all items and for the total score, female scores were significantly elevated compared to males.

### Integration of variables

**Figure 1:**
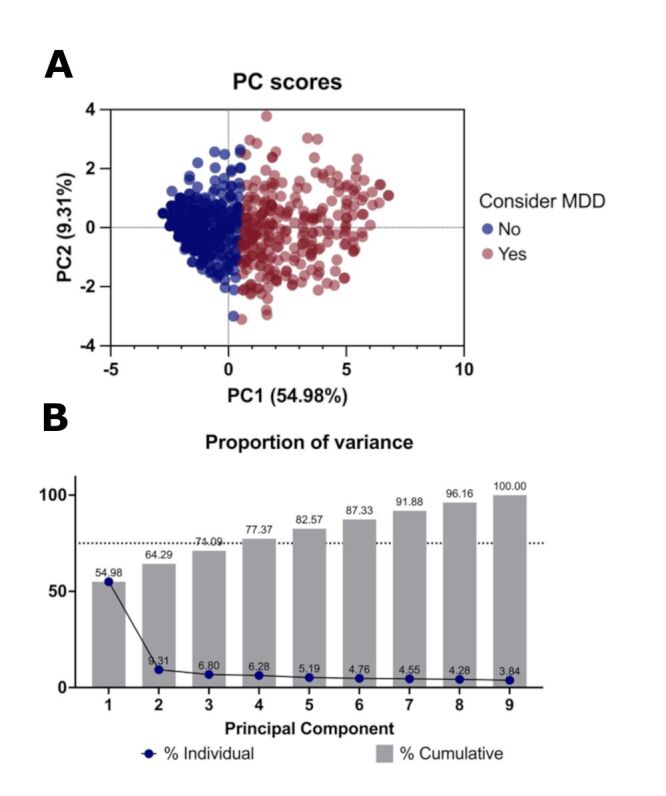
Results of the Principal Component Analysis. **Panel A**. Biplot representing both the score plot and the loading plot. Blue points represent the group whose PHQ-9 score is not suggestive of MDD, and red points represent the group in which MDD must be considered. **Panel B**. Scree plots reporting the percentage of variance explained by each factor and the eigenvalues, where the first principal component has a proportion of variance of 54.98% and an eigenvalue of 4.95. MDD – major depressive disorder, PHQ9 – 9-item Patient Health Questionnaire.

Principal Component Analysis (PCA)was applied to the present data to determine the factor structure of the PHQ-9 in the present sample population. Initial eigenvalues indicated that the first factor explained 54.98% of the variance, with PHQ-9 items two (feeling down, depressed, hopeless), six (feeling bad about self, feelings of failure), and five (appetite changes) showing the largest association with this factor (loading scores being 0.796, 0.786, and 0.780, respectively). Interestingly, suicidal ideation (item nine) showed the lowest association with this factor, while also showing a comparable association with factor two, suggesting that suicidal ideation may have latent characteristics distinct from the other items. Items two, six, and eight also showed low positive associations with factor two (**Figure 1**, **Table 3**).

**Table 3:** PC loading scores for each of the PHQ-9 items

PHQ-9 Item	PC1	PC2	PC3	PC4
1 (lack of interest)	0.708	−0.317	0.266	−0.359
2 (hopelessness)	0.796	0.210	0.237	−0.090
3 (sleep disturbance)	0.700	−0.420	0.126	0.428
4 (tiredness)	0.760	−0.312	−0.053	−0.084
5 (appetite changes)	0.780	−0.064	0.057	0.299
6 (feelings of failure)	0.786	0.234	0.055	−0.216
7 (difficulty concentrating)	0.745	−0.096	−0.435	−0.241
8 (motoric alterations)	0.717	0.217	−0.471	0.169
9 (suicidal ideation)	0.671	0.552	0.221	0.125

PC – principal component, PHQ-9 – 9-item Patient Health Questionnaire

## DISCUSSION

This study highlights the elevated prevalence of depressive symptoms among adolescents in rural communities of San Luis Potosí, Mexico. Over half of the participants reported experiencing depressive symptoms, with 23% meeting the criteria for a probable diagnosis of MDD. We observed significant gender differences in the scores of all individual PHQ-9 items, although in general the statistical effect of gender on measures of depression (PHQ-9 scores, probable MDD diagnosis) was small. Familial antecedents of depression (as reported by the participants) were most strongly associated with more severe depressive symptoms and having past episodes of depression, perhaps indicating a more chronic disease course in these subjects. Fulfilling the criteria for a probable diagnosis of MDD was moderately associated with having past episodes of depression and strongly associated with more frequent thoughts of self-harm and suicide. The PCA revealed a single factor that explained a majority (55%) of the observed variance, with feeling depressed and hopeless, feeling bad about oneself, and appetite changes showing the highest associations with this factor. A second factor explained an additional 10% of the variance, with suicidal ideation being the only item loading highly on this factor.

Published data on the prevalence of depression in Mexican adolescents is rather sparse. The prevalence of borderline depression in adolescent women (age 15–19) of a rural indigenous Mexican community was reported to be 11% [32]. In adolescents of indigenous communities in the Mexican state of Chiapas, the prevalence of mild to severe symptoms of depression was 20.6% [23]. Elliot and colleagues [33] reported the prevalence of clinical depression in young adults (18–29 years old) of rural communities in the state of Chiapas to be 6.2%, with women showing a higher prevalence than men (6.8% compared to 4.7%). Using another method of survey, the Center for Epidemiological Studies Depression Scale (CES-D), students of 12–15 years old in Mexico City and in the state of Michoacan, the prevalence of clinical symptoms of depression was 12.1% (17.8% of female subjects, 7.7% of male subjects), and the prevalence of subclinical depression was determined to be 27.3%; this prevalence was approximately equal for female and male subjects [14]. The present results indicate a notably higher prevalence and severity of depressive symptoms than have been previously reported (23% of the present sample fulfilled criteria for a probable MDD diagnosis), although direct comparisons are problematic due to differences among studies with respect to the survey methods that were applied, criteria used to determine and report symptom severity, the age of subjects, and socioeconomic and cultural differences among study populations. Therefore, it is crucial for future research efforts to adapt uniform methodologies for surveying adolescent populations and apply these standardised methodologies in different regions (urban, marginalised urban, rural, indigenous, etc.), in order to identify common risk factors as well as those unique to specific cultural and socioeconomic contexts.

A large body of literature indicates that depressive symptoms are more prevalent and severe in women, and that this gender difference manifests in early adolescence. Undoubtedly, this gender difference has both social and physiological underpinnings [34,35]. In the present sample of adolescents and young adults, females exhibited significantly higher scores on most PHQ-9 items, with the largest statistical effect of gender on responses to PHQ-9 items of feeling depressed and hopeless, alterations in sleep, and changes in appetite. Similarly, female subjects more frequently reported having familial antecedents of depression, prior episodes of depression, more frequent thoughts of self-harm, and were more likely to fulfil criteria for a probable diagnosis of MDD. However, it is notable that for most comparisons, the statistical effect size of gender was rather small (<0.25). This result underscores the need to study risk factors for adolescent depression that are unique to each gender, as well as those common to both, and apply this knowledge to the development of programmes for preventative care.

A significant proportion of subjects (52%) reported that they had experienced a depressive episode in the past. Of these subjects, 34% fulfilled criteria for a probable diagnosis of MDD at the time of the survey, compared to 10% of the subjects that reported they had not previously experienced a depressive episode. This result indicates that questioning adolescents about past depressive episodes might serve as a useful predictor of future clinical depression, and that psychoeducation sessions on how to recognise symptoms of depression (past or present episodes) may help channel vulnerable subjects that may not be currently experiencing a depressive episode, into preventative care. Early detection and treatment of depression in adolescents is critical: a study by Benjet and colleagues reported that 7% of adults aged 18–64 had suffered from an episode of major depression at least once in their lifetime; of these, 27% had their first episode before the age of 18 [36]. Considering that in Mexico depression accounts for 2.1 and 6.7% of disability adjusted life years for men and women, respectively [5], it is critical to identify and treat symptoms early in life to prevent future incapacity.

The results of our study indicated that familial antecedents of depressive symptoms and family dynamics should be an area focus for future research. Thus, 28% of the participants reported a family member with depressive symptoms – most frequently the mother – and that having a female primary caregiver was associated with slightly elevated PHQ-9 scores. Familial antecedents of depression were also significantly associated with increased thoughts of self-harm and a probable diagnosis of MDD: a family history of depression was associated with an almost doubling of the proportion of subjects that fulfilled criteria for probable diagnosis of MDD (33 *vs*. 19%). This difference is consistent with the relationship between parental MDD and offspring MDD that was previously reported (26% of subjects who had one parent that suffered from MDD were also diagnosed with MDD, compared to 12% of subjects that did not have a parent with MDD) [37]. The relationship between depressive symptoms and familial antecedents of depression likely has both genetic and non-genetic underpinnings. Familial transmission of mental illness requires family-based approaches in mental health interventions, particularly in regions where systemic support is limited.

A recent meta-analysis of 90 confirmatory factor analyses from 40 different countries indicated either a 1- or 2-factor structure of the PHQ-9 [38]. The 1-factor solution comprised all nine items (or items 1–8, omitting item 9), while the 2-factor solution most frequently comprised a ‘somatic’ factor (comprising items 3, 4 and 5) and a ‘nonsomatic/cognitive-affective’ factor (items 1, 2, 6–9). The present PCA results indicate that a single factor explains a majority of the variance and appears to be most associated with a probable MDD diagnosis. Feeling depressed and hopeless, feelings of failure, and appetite changes were most highly associated with this factor. Nevertheless, it is notable that suicidal ideation was moderately associated with both the first and second factors, while feeling depressed and hopeless, feelings of failure, and activity changes also showed positive (albeit low) associations with the second factor. Similarly, a recent latent class analysis of suicidal behaviour of Mexican adolescents indicated two populations of adolescents that engaged in suicidal behaviour: one population (12% of the adolescent sample) in which suicidal behaviour was associated with major depressive disorder, and a separate population (8% of adolescents) in which suicidal behaviour was not associated with clinical depression [39]. In line with this proposal, Casas-Muñoz and colleagues [40] identified that behavioural, somatic, oppositional-defiant, and anxiety problems were significant predictors of suicidal behaviour, independently of depression. Taken together, these findings underscore the importance of studying suicidal ideation and suicidal behaviour as distinct phenomena that may not always be associated with clinical depression. Thus, suicidal ideation and behaviour demand specialised attention and preventative strategies and may be influenced by unique contextual, cultural, and gender-related factors.

Studies from around the world have identified several risk factors for adolescent depression, including bullying and cyberbullying [41], an unhealthy diet high in processed foods and low in grains, fruits and vegetables [42,43], sedentary behaviour [44], and problematic smartphone use [45]. Studies of Mexican adolescents have associated suicidal ideation and suicidal behaviour with violent crime victimisation [46], bullying and poor family functioning [47,48], as well as genetic risk factors [49]. A survey of Mexican adolescents revealed that the prevalence of depression was lowest (5%) in adolescents that were enrolled in school, and highest (12%) in those that were not in school and either working or not employed [50]. However, the population of Mexico is incredibly diverse, with socioeconomic and cultural characteristics unique to each geographical region. In order to design and implement effective regional and community-based programmes to promote mental health, it will be necessary to collect data on depressive symptoms and associated risk factors in the communities that are to be served. As we have demonstrated in the present study, such data can be collected as part of a psychoeducation and mental health screening programme.

A psychoeducation and mental health screening programme such as the one applied in the present study here can identify vulnerable individuals based on their PHQ-9 scores, who then could be channelled into preventative intervention programmes. Preventive interventions should first prioritise cognitive skill enhancement, which is both malleable and capable of mitigating the impact of early adverse experiences, particularly in marginalised settings [51,52]. Such intervention should identify and address key individual vulnerabilities, such as low self-esteem, diminished optimism, poor emotional control, or poor stress coping mechanisms [53–56]. Healthy family dynamics, including parental support and positive communication significantly contribute as protective factors and should also be considered within preventative treatment [48,51]. Positive school environments and supportive teacher relationships should be encouraged as these can act as important buffers against depression [50]. Psychoeducation within the community and school settings can help adolescents identify their own depressive symptoms, reduce the stigma of mental illness and encourage treatment. Psychoeducation efforts should also emphasise lifestyle changes that may support mental health, including a healthy diet, exercise, regular sleep, and reduced use of smartphones. Education on the importance of sufficient and regular sleep may be particularly important, as the present data indicate that almost a third of the subjects reported significant sleep problems and fatigue. At a more global level, prevention efforts should address systemic issues such as poverty, gender inequality, and lack of access to education and health care.

### Limitations

This study is limited by its reliance on self-reported measures, which may introduce biases. Additionally, cross-sectional design prevents the identification of causal relationships or the assessment of symptom trajectories over time – an important consideration for future research on the course of depressive symptoms in this population. Although every effort was made to obtain a representative and unbiased population sample (recruitment of participants was carried out in several different community contexts), the nature of the recruitment process, in particular recruitment in the context of psychoeducation, may have resulted in over-representation of participants that suffer from depression. We also cannot rule out the possibility that the psychoeducation sessions administered prior to the application of the PHQ-9 may have influenced participants' responses, potentially introducing a priming effect by increasing symptom awareness or openness in reporting. Furthermore, although 1058 adolescents and young adults were invited to participate, approximately 250 did not complete the PHQ-9 due to either declining participation or submitting incomplete responses, often related to time constraints or other situations like personal discomfort with the questionnaire content. While no significant demographic differences were observed between completers and non-completers, the potential for response bias remains and should be considered when interpreting the results. Finally, future research should prioritise longitudinal designs to explore causal pathways and symptom trajectories, and qualitative studies to capture the lived experiences of indigenous and rural adolescents.

## CONCLUSIONS

The high prevalence of depressive symptoms in this study calls for urgent public health interventions tailored to underserved rural contexts. These findings are consistent with regional data suggesting that social determinants play a more critical role in Latin America's elevated depression prevalence compared to global averages [57]. Tackling these determinants requires integrated policies that bridge health, education, and social welfare systems.

The study provides valuable insights into the mental health challenges faced by adolescents in rural and indigenous communities in Mexico. The findings support the implementation of culturally adapted, community-based psychoeducation, screening and treatment programmes, ideally delivered in local languages and incorporating indigenous cultural frameworks. Digital tools for early mental health screening, as proposed by Martínez-Nicolás [58], hold promise but require equitable access strategies to ensure inclusion of adolescents from marginalised backgrounds without reliable internet connectivity. With almost one quarter of participants of the present study meeting criteria for a probable diagnosis of MDD, it is imperative to prioritise targeted mental health services and culturally relevant interventions. In the future, addressing the intersection of gender, socioeconomic status, and indigenous identity is critical for reducing mental health disparities and improving outcomes in these vulnerable populations.
